# Bile Acid Profiling Reveals Distinct Signatures in Undernourished Children with Environmental Enteric Dysfunction

**DOI:** 10.1093/jn/nxab321

**Published:** 2021-10-27

**Authors:** Xueheng Zhao, Kenneth D R Setchell, Rong Huang, Indika Mallawaarachchi, Lubaina Ehsan, Edward Dobrzykowski III, Junfang Zhao, Sana Syed, Jennie Z Ma, Najeeha T Iqbal, Junaid Iqbal, Kamran Sadiq, Sheraz Ahmed, Yael Haberman, Lee A Denson, Syed Asad Ali, Sean R Moore

**Affiliations:** Division of Pathology & Laboratory Medicine, Cincinnati Children's Hospital Medical Center, Cincinnati, OH, USA; Division of Pathology & Laboratory Medicine, Cincinnati Children's Hospital Medical Center, Cincinnati, OH, USA; Department of Pediatrics, University of Cincinnati College of Medicine, Cincinnati, OH, USA; Division of Pathology & Laboratory Medicine, Cincinnati Children's Hospital Medical Center, Cincinnati, OH, USA; Department of Public Health Sciences, University of Virginia, Charlottesville, VA, USA; Division of Pediatric Gastroenterology, Hepatology, and Nutrition, Department of Pediatrics, University of Virginia, Charlottesville, VA, USA; Division of Pathology & Laboratory Medicine, Cincinnati Children's Hospital Medical Center, Cincinnati, OH, USA; Division of Human Genetics, Cincinnati Children's Hospital Medical Center, Cincinnati, OH, USA; Division of Pathology & Laboratory Medicine, Cincinnati Children's Hospital Medical Center, Cincinnati, OH, USA; Division of Pediatric Gastroenterology, Hepatology, and Nutrition, Department of Pediatrics, University of Virginia, Charlottesville, VA, USA; Departments of Pediatrics and Child Health, Aga Khan University, Karachi, Pakistan; Department of Public Health Sciences, University of Virginia, Charlottesville, VA, USA; Departments of Pediatrics and Child Health, Aga Khan University, Karachi, Pakistan; Departments of Biological and Biomedical Sciences, Aga Khan University, Karachi, Pakistan; Departments of Pediatrics and Child Health, Aga Khan University, Karachi, Pakistan; Departments of Biological and Biomedical Sciences, Aga Khan University, Karachi, Pakistan; Departments of Pediatrics and Child Health, Aga Khan University, Karachi, Pakistan; Departments of Pediatrics and Child Health, Aga Khan University, Karachi, Pakistan; Department of Pediatrics, University of Cincinnati College of Medicine, Cincinnati, OH, USA; Department of Pediatrics, Sheba Medical Center, Tel-HaShomer, affiliated with the Tel-Aviv University, Israel; Division of Gastroenterology, Hepatology, and Nutrition, Cincinnati Children's Hospital Medical Center, Cincinnati, OH, USA; Department of Pediatrics, University of Cincinnati College of Medicine, Cincinnati, OH, USA; Division of Gastroenterology, Hepatology, and Nutrition, Cincinnati Children's Hospital Medical Center, Cincinnati, OH, USA; Departments of Pediatrics and Child Health, Aga Khan University, Karachi, Pakistan; Division of Pediatric Gastroenterology, Hepatology, and Nutrition, Department of Pediatrics, University of Virginia, Charlottesville, VA, USA

**Keywords:** environmental enteric dysfunction (EED), bile acid, biomarkers, stunting, undernutrition, UHPLC-MS/MS, endoscopy

## Abstract

**Background:**

Intestinal inflammation and malabsorption in environmental enteric dysfunction (EED) are associated with early childhood growth faltering in impoverished settings worldwide.

**Objectives:**

The goal of this study was to identify candidate biomarkers associated with inflammation, EED histology, and as predictors of later growth outcomes by focusing on the liver-gut axis by investigating the bile acid metabolome.

**Methods:**

Undernourished rural Pakistani infants (*n* = 365) with weight-for-height Z score (WHZ) < –2 were followed up to the age of 24 mo and monitored for growth, infections, and EED. Well-nourished local children (*n* = 51) were controls, based on consistent WHZ > 0 and height-for-age Z score (HAZ) > –1 on 2 consecutive visits at 3 and 6 mo. Serum bile acid (sBA) profiles were measured by tandem MS at the ages of 3–6 and 9 mo and before nutritional intervention. Biopsies and duodenal aspirates were obtained following upper gastrointestinal endoscopy from a subset of children (*n* = 63) that responded poorly to nutritional intervention. BA composition in paired plasma and duodenal aspirates was compared based on the severity of EED histopathological scores and correlated to clinical and growth outcomes.

**Results:**

Remarkably, >70% of undernourished Pakistani infants displayed elevated sBA concentrations consistent with subclinical cholestasis. Serum glycocholic acid (GCA) correlated with linear growth faltering (HAZ, r = –0.252 and –0.295 at the age of 3–6 and 9 mo, respectively, *P* <0.001) and biomarkers of inflammation. The proportion of GCA positively correlated with EED severity for both plasma (r_s_ = 0.324 *P* = 0.02) and duodenal aspirates (r_s_ = 0.307 *P* = 0.06) in children with refractory wasting that underwent endoscopy, and the proportion of secondary BA was low in both undernourished and EED children.

**Conclusions:**

Dysregulated bile acid metabolism is associated with growth faltering and EED severity in undernourished children. Restoration of intestinal BA homeostasis may offer a novel therapeutic target for undernutrition in children with EED. This trial was registered at clinicaltrials.gov as NCT03588013.

## Introduction

Environmental enteric dysfunction (EED; also known as environmental enteropathy) is a major impediment to achieving global targets for reducing childhood undernutrition (1). Undernutrition impacts ∼22% of children aged <5 y globally and underlies almost half of all deaths in this age group (2). EED is also associated with an increased risk of morbidity, stunting, and reduced oral vaccine immunogenicity in impoverished settings (3), where inadequate water, hygiene, and sanitation are considered critical EED risk factors (4, 5). Children fed marginal diets and exposed repeatedly to enteropathogens develop a chronic enteropathy, with altered structure and function of the small intestine. Chronic mucosal inflammation, villus blunting, and increased permeability, with reduced absorptive capacity of the small intestine, are the primary sequelae described in EED patients (6). EED is also implicated in micronutrient deficiency and intestinal antigen translocation (7, 8). However, the underlying mechanisms and pathogenesis of EED remain poorly understood. Dietary interventions and water, sanitation, and hygiene measures, alone or in combination, are often ineffective and fail to substantially ameliorate EED and its associated stunting (3, 9). Furthermore, current EED biomarkers fail to capture the full spectrum of EED and links to metabolism (10).

Emerging evidence from sub-Saharan Africa suggests bile acid (BA) homeostasis is disrupted in children aged >1 y with either EED and/or severe acute malnutrition (SAM) (11, 12). BA function as detergents in the intestine to facilitate lipid digestion and absorption. In the liver, bile acids play a critical role in providing the driving force for stimulating bile flow while also functioning as key signaling molecules that regulate numerous metabolic pathways associated with childhood growth (12, 13). Importantly, animal studies show BA regulate intestinal inflammation by modulating gut regulatory T cell homeostasis (14–16). Restoration of the intestinal BA pool was shown to ameliorate host susceptibility to inflammation (14). Furthermore, BA mediate colonization and maturation of the newborn gut microbiota (17). Key findings of immaturity of fecal microbiota in stunted children (18) and a causal role of the small intestinal microbiota in EED propel current efforts to develop microbiome-directed interventions against EED (19).

As part of the Study of Environmental Enteropathy and Malnutrition (SEEM)-Pakistan, we measured the serum BA metabolome in a large cohort of rural Pakistani children followed longitudinally from the age of 3–24 mo for growth and infection and biological samples were collected between the age of 3–6 mo, and again at the age of 9 mo (2, 20). A subset of this cohort, refractory to multiple nutritional interventions, underwent esophagogastroduodenoscopy (EGD) to evaluate enteropathies. Uniquely, paired samples of duodenal aspirates and plasma collected at the time of endoscopy permitted us to determine the extent to which plasma bile acid composition reflects intraluminal duodenal BA in EED. The key findings of this study reveal, *1*) persistent elevated plasma BA concentrations, consistent with cholestasis are a feature in a high proportion of children with growth failure, *2*) specifically, elevated serum glycocholic acid (GCA) concentrations correlate with the severity of biomarkers of inflammation and can predict linear growth faltering, and *3*) a unique BA signature of increased proportions of GCA and depleted secondary BA in plasma and duodenal aspirates occurs in children with EED refractory to nutritional intervention.

## Methods

### Study design and specimen collection

SEEM in Pakistan is a prospective study that enrolled children at birth in Matiari, Pakistan, a region with high prevalence of undernutrition, between 2016 and 2019 (2). This trial was registered at clinicaltrials.gov as NCT03588013.

Study participants comprised a local healthy child control group (*n* = 51) and a group of undernourished children (*n* = 365) that were evaluated for symptoms of EED and followed for growth up to the age of 24 mo. Undernourished children were defined by a weight-for-height Z score (WHZ) < –2 at enrollment, whereas the healthy control cohort was based on consistent WHZ >0 and a height-for-age Z score (HAZ) > –1 on 2 consecutive visits between the ages of 3 and 6 mo prior to nutritional intervention. Linear and ponderal growth for each child was monitored monthly. Blood, urine, and feces were collected at the age of 3–6 mo and 9 mo, respectively. After enrollment, the parents/caregivers of all participants underwent a series of educational programs to improve the child's nutritional status. Upon enrollment, participants received a 4-wk home-delivered educational program that focused on breastfeeding and complementary feeding. Counseling was performed by the study staff using standardized teaching materials. If the WHZ score remained < −2 by the age of 9 mo, despite the initial educational counseling, families were shown a 10 min educational video fortnightly detailing the best practices for complementary feeding and compliance to the instructions was recorded during weekly home visits. Nutritional intervention was given to 189 children who had a WHZ score < −2 at the age of 9–12 mo using high calorie AchaMum supplementary food containing protein and essential fatty acids. Among the undernourished children who responded poorly to nutritional intervention, in total 63 were evaluated by EGD at the Aga Khan University Hospital (AKUH). Biopsy specimens were obtained from these children for detailed assessment of the histopathology of EED (**Supplemental Table 1**). Other poorly responding children (*n* = 8) declined EGD. Given the ethical limitation and lack of clinical justification for performing EGD on adequately growing children, it was not possible to collect EGD samples from local Matiari controls. Nevertheless, as a compromise, after evaluating the histology, the biopsied group was divided into those children above the 50th percentile and those below the 50th percentile based on the biopsy scoring system (**Supplemental Table 2**). Paired plasma and duodenal aspirate samples were collected at the time of endoscopy while these children were sedated and in a fasted state. Presaline lavage duodenal aspirates were attempted and collected. It was not always possible to collect samples from all the children at every time point, however, a comprehensive sample size was obtained. The study design and sample collection timeline are outlined in **Supplemental Figure 1**.

### BA profiling using ultra-performance LC coupled with tandem MS

The serum and plasma BA metabolome was determined by stable-isotope dilution analysis using a fully validated LC-electrospray ionization-MS (LC-ESI-MS) method (**Supplemental Methods**). In total, the 15 major primary and secondary bile acids and their conjugates were measured, which included cholic acid (CA), chenodeoxycholic acid (CDCA), deoxycholic acid (DCA), lithocholic acid (LCA), glycocholic acid (GCA), glycochenodeoxycholic acid (GCDCA), glycodeoxycholic acid (GDCA), glycolithocholic acid (GLCA), glycoursodeoxycholic acid (GUDCA), taurocholic acid (TCA), taurochenodeoxycholic acid (TCDCA), taurodeoxycholic acid (TDCA), taurolithocholic acid (TLCA), tauroursodeoxycholic acid (TUDCA), and ursodeoxycholic acid (UDCA). The lower limit of quantification (LLOQ) was 0.1 μg/mL for the 15 BA measured. All samples were analyzed at a single site (Cincinnati Children's Hospital Medical Center, Cincinnati, OH, USA) according to a standard operating procedure (SOP # PATH.CMS.1065). The facility is accredited with the College of American Pathologists (CAP license #1,667,801) and with Clinical Laboratory Improvement Amendments certification (CLIA 88 license #36D0656333). Duodenal aspirate samples were extracted using solid phase extraction (SPE) (21). Serum and plasma bile acid concentrations are expressed as μmol/L and the total bile acid concentration is represented by the sum of the individual bile acid species measured. The duodenal bile acid composition is expressed as percent composition of the individual bile acid species. The percentage of primary and secondary BA was calculated as the ratio of the sum of the concentrations of CDCA and CA to the sum of the concentrations of DCA, LCA, and UDCA in all their conjugate forms.

### Determination of serum 7α-hydroxy-4-cholesten-3-one by tandem MS

7α-hydroxy-4-cholesten-3-one (sterol-C4) was measured in plasma using a fully validated stable-isotope dilution LC-ESI-MS assay according to a proprietary assay developed at Cincinnati Children's Hospital Medical Center (CCHMC) (SOP # PATH.CMS.1046).

### Hydrophobicity index of bile acid pool

The hydrophobicity index (HI) of the BA pool of plasma and duodenal aspirate was calculated according to the relative contributions of the individual BA to the total BA pool and their HIs (22).

### Histopathology

Features of EGD biopsy tissue samples were comprehensively assessed by a team of pathologists at Aga Khan University Hospital (AKUH) using an EED scoring system developed by the Environmental Enteric Dysfunction Biopsy Initiative (EEDBI) Consortium. Histological severity of EED was evaluated using this biopsy scoring system (23) and score criteria are described in detail in Supplemental Table 1.

### Investigated clinical biomarkers of EED

Lactose/rhamnose (L/R) dual sugar permeability testing was conducted and validated based on EEDBI Consortium protocols. In those children who underwent endoscopy at AKUH, fecal calorimetry (6200 Isoperibol Calorimeter; Parr Instrument Company) was used to obtain total fecal energy content (24). Blood cytokine/chemokine assays used the MILLIPLEX MAP Human Cytokine/Chemokine panel (EMD Millipore Corporation). α-1 acid glycoprotein (AGP) or orosomucoid, ferritin, and C-reactive protein (CRP) were analyzed on an automated biochemistry analyzer, Roche/Hitachi 902, and insulin-like growth factor (IGF-I) was analyzed using a LIAISON Diasorin. All other clinical markers in urine and fecal EED biomarkers were measured at the age of 3–6 and 9 mo by commercial assay kits as described in detail previously (25).

### Statistical analysis

To determine the association of BA with growth, we assessed the relation between serum BA composition at the age of 9 mo with WHZ and weight-for-age Z score (WAZ) scores at the age of 24 mo. Conditional random forests (CRF) analysis was performed to evaluate the relative importance of initial anthropometry parameters and bile acids accounting for their correlations with a threshold of ≥0.5. The top 5 predictors from CRF were further evaluated in a linear regression model. BA compositions were either log transformed (GUDCA, TCA, GCA) or dichotomized as 0 compared with >0 (GDCA, TDCA, DCA, UDCA, LCA) due to their skewed distributions. Statistical tests were conducted with a 2-sided α level of 0.05. In the case of comparison of subgroups of Pakistani EED children, slightly less than significant *P* was also noted to indicate the trend. For explorative analysis, Pearson's correlation was conducted for continuous variables including an association between BA composition in plasma and duodenal aspirate, and HAZ. Spearman's rank correlation was performed to examine the relation between BA biomarkers and EED histopathological scores. Due to the explorative nature, no adjustment was performed in these correlation analyses except for the primary anthropometric response at 24 mo. Dispersion of data was reported as mean ± SEM or 95% CI unless indicated otherwise. All statistical analysis was conducted in R Language and Environment for Statistical Computing (www.r-project.org) or SAS (version 9.4, SAS Institute) statistical software.

### Ethics and study approval

Approval for this study was obtained from CCHMC (Study ID 2016–0387) and AKUH Ethical Review Committee (3836-Ped-ERC-15). Written informed consent was obtained from the parents/guardians of the children enrolled.

## Results

### Clinical cohorts

Participants of the study all resided in the rural district of Matiari, Pakistan, and were enrolled from birth and followed up to the age of 2 y (2). Among this cohort of 416 infants, 365 children aged between 3–6 mo were assigned to the undernourished group and 51 children comprised the healthy nourished control group. Nutrition interventions for children with wasting at 9 mo (WHZ < -2) consisted of educational and supplementation programs lasting 2 mo, with monthly anthropometric measurements collected. In total, 63 children failed to respond to nutritional intervention by the age of 12–24 mo and subsequently underwent EGD to diagnose underlying EED. The demographics, growth measurements, and clinical characteristics of the enrolled children at different time points are summarized in   **Tables 1** and **2**.

**TABLE 1:** Demographics and anthropometrics of Pakistani undernourished and healthy children^1^

	Healthy control (WHZ >0, HAZ>–1 at enrollment)	Undernourished children (WHZ < -2 at enrollment)		EGD children (WHZ < -2 after nutritional intervention and biopsy sample collected)	
N	51	365		63	
Female, %	24 (47%)	142 (39%)		18 (29%)	
Age, m	3–6	9	3–6	9	1.60 (1.52, 1.67)
HAZ	−0.665 (–0.951, –0.379)	–0.905 (–1.238, –0.572)	–2.361 (–2.515, –2.207)	–2.504 (–2.664, –2.344)	–3.12 (–3.43, –2.82)
WAZ	0.699 (0.443, 0.955)^1^	0.638 (0.381, 0.896)	–2.63 (–2.70, –2.56)	–2.04 (–2.16, –1.91)	–3.21 (–3.45, –2.98)

1 Data are represented as mean and 95% CI. EGD, esophagogastroduodenoscopy; HAZ, height-for-age Z score; WAZ, weight-for-age Z score; WHZ, weight-for-height Z score.

**TABLE 2:** Investigated EED biomarkers of Pakistani undernourished and healthy children at 2 study time points^1^

	Healthy control (WHZ >0, HAZ>–1 at enrollment) *n* = 51		Undernourished children (WHZ < -2 at enrollment) *n* = 365	
Age, mo	3–6	9	3–6	9
GLP, pg/mL	1210 (1010, 1420)	2070 (1550, 2550)	1350 (1220, 1480)	1380 (1240, 1520) ^2^
Leptin, pg/mL	408 (363, 454)	305 (259, 350)	173 (158, 187)^3^	221 (196, 245) ^2^
Ferritin, ng/mL	63.5 (24.9, 102)	22.6 (11.7, 33.5)	136 (122, 150)^3^	41.0 (30.6, 51.4)^4^
Prealbumin, mg/dL	15.8 (14.5, 17.0)	15.8 (14.6, 16.9)	14.0 (13.6, 14.4)^4^	14.5 (14.1, 14.9)^4^
AGP, mg/dL	104 (92.4, 115)	99.0 (88.5, 109)	103 (96.7, 110)	112 (107, 117)^4^
CRP, mg/dL	0.360 (0.173, 0.546)	0.219 (0.132, 0.306)	0.481 (0.358, 0.604)	0.633 (0.347, 0.919) ^2^
IGF-I, ng/mL	39.4 (32.0, 46.8)	30.6 (26.0, 35.1)	26.6 (24.6, 28.5) ^2^	23.6 (21.6, 25.7) ^2^
HGB, g/L	10.8 (10.5, 11.2)	10.8 (10.5, 11.2)	10.4 (10.3, 10.6)^4^	10.4 (10.2, 10.5)^4^
Rota_IgA, U/mL	186 (10.5, 362)	256 (91.4, 420)	94.2 (29.4, 159)	447 (102, 792)
IP-10, pg/mL	3080 (2340, 3820)	2260 (1730, 2780)	2544 (2330, 2760)	2420 (2150, 2690)
IL-10, pg/mL	73.1 (37.7, 109)	50.4 (20.1, 80.8)	197 (154, 239)^3^	186 (144, 229)^3^
INF-γ, pg/mL	17.2 (7.55, 26.9)	19.0 (–0.50, 38.6)	130 (92.0, 167)^3^	148 (99.3, 196)^3^
IL-8, pg/mL	34.0 (10.5, 57.5)	36.8 (17.4, 56.2)	130.8 (98.2, 163)^3^	133 (101, 165)^3^
IL-1β, pg/mL	5.45 (2.70, 8.20)	3.98 (2.55, 5.42)	38.3 (24.2, 52.4)^3^	42.4 (21.1, 63.8)^3^
IL-6, pg/mL	9.47 (4.02, 14.9)	7.26 (4.43, 10.1)	55.1 (34.1, 76.0)^3^	83.2 (50.8, 115.6)^3^
IL-12, pg/mL	6.03 (2.61, 9.45)	3.95 (1.94, 5.96)	39.9 (18.3, 61.5) ^2^	60.6 (26.1, 95.0) ^2^
TNF-α, pg/mL	91.1 (47.5, 135)	73.6 (53.9, 93.2)	216 (173, 259)^3^	207 (163, 250)^3^
MCP-1, pg/mL	917 (779, 1056)	960 (800, 1120)	1250 (1160, 1330)^3^	1083 (993, 1170)
Urine claudin-15, ng/mL	0.81 (0.69, 0.93)	0.89 (0.80, 0.97)	1.65 (1.51, 1.78)^3^	1.93 (1.77, 2.09)^3^
Urine creatinine, μmol/L	168 (141, 194)	197 (152, 242)	131 (119, 143)^4^	178 (161, 195)
Fecal myeloperoxidase, ng/mL	19,100 (8270, 30,021)	13,600 (3760, 23,400)	16,400 (12,070, 20,820)	9960 (7770, 12,200)
Fecal neopterin, nmol/L	2260 (1730, 2780)	2530 (1610, 3440)	2220 (2014, 2420)	2270 (2059, 2490)

1 Investigated EED biomarkers were measured in blood unless noted otherwise. Data are represented as mean and 95% CI. Difference in each characteristic were evaluated by student's t-test. AGP, α-1 acid glycoprotein; CRP, C-reactive protein; EED, environmental enteric dysfunction; HAZ, height-for-age Z score; GLP, glucagon-like peptide 2; HGB, hemoglobin; IGF-I, insulin-like growth factor I; INF-γ, interferon-γ; IP-10, interferon-inducible protein 10; MCP-1, monocyte chemoattractant protein 1; WHZ, weight-for-height Z score.

2 *P* <0.01 compared with the healthy control group at the corresponding time point.

3 *P* <0.001 compared with the healthy control group at the corresponding time point.

4 *P* <0.05 compared with the healthy control group at the corresponding time point.

### Serum BA profiles at the age of 3–6 and 9 mo in Pakistani children

**FIGURE 1:**
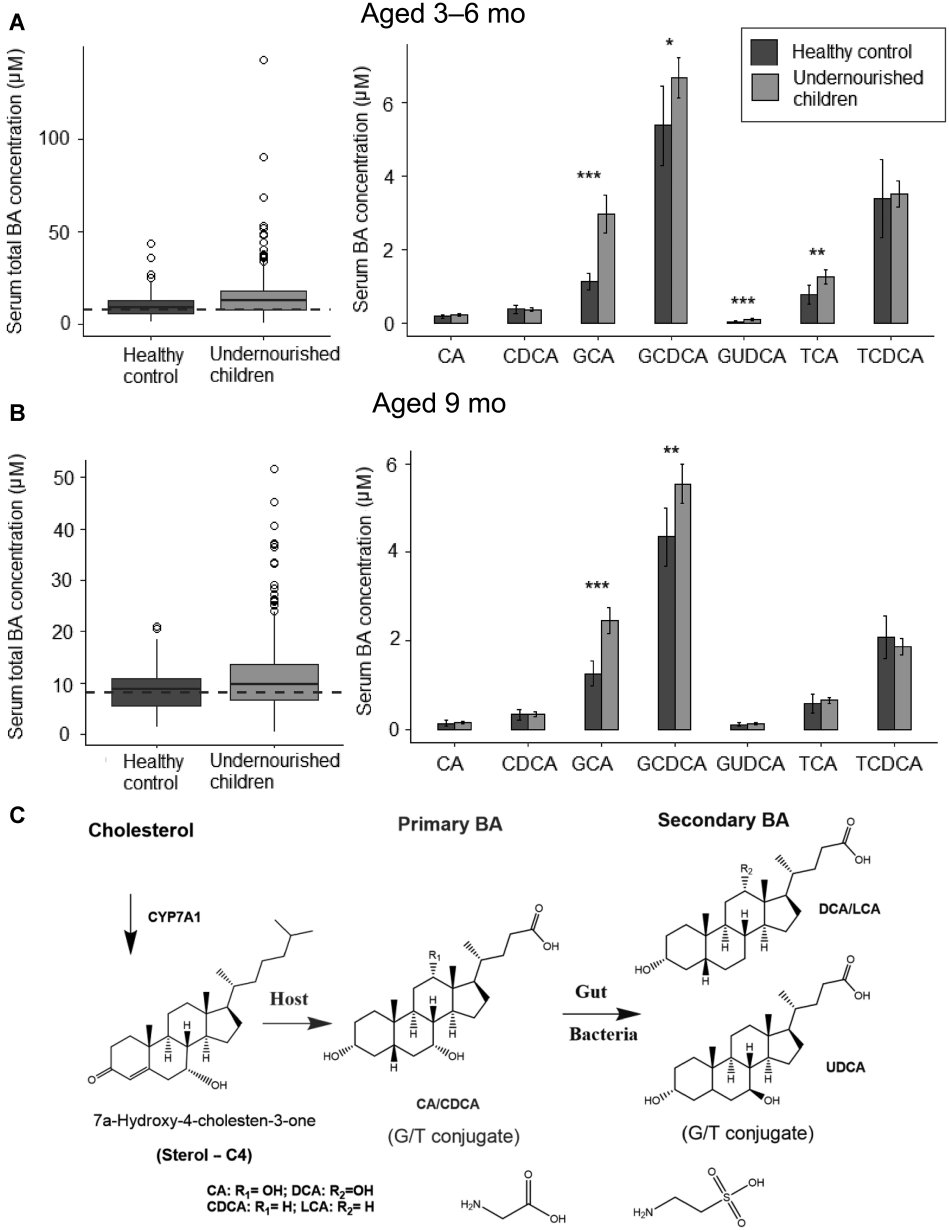
Serum BA profiles in Pakistani undernourished and healthy children aged between 3 and 6 mo, and 9 mo. (A–B) Serum BA profile in Pakistani undernourished children and healthy controls aged 3–6 (*n* = 339 undernourished and 50 healthy controls) and 9 (*n* = 295 undernourished and 47 healthy controls) mo. On the left, total BA profiles were plotted (Dashed line indicates 8 μM an upper limit of normal range), and on the right 7 BA subspecies (out of 15 quantified BA) with >1% of the total BA pool plotted. *P <0.05, **P <0.01, ***P <0.001. (C) Scheme of biosynthesis pathway and metabolic transformations of BA in humans. BA, bile acid; CA, cholic acid; CDCA, chenodeoxycholic acid; DCA, deoxycholic acid; G, glycine; GCA, glycocholic acid; GCDCA, glycochenodeoxycholic acid; GUDCA, glycoursodeoxycholic acid; LCA, lithocholic acid; T, taurine; TCA, taurocholic acid; TCDCA, taurochenodeoxycholic acid; UDCA, ursodeoxycholic acid.

Serum BA profiles were quantified for the principal primary and secondary bile acids at the age of 3–6 and 9 mo and prior to nutritional intervention in order to study the BA profiles with EED biomarkers. A total of 730 serum samples were analyzed from children over these 2 time points. Of these, 680 serum samples were paired for both age groups and the majority of the BA detected in serum of both the undernourished and well-nourished Pakistani children were conjugated with glycine and taurine (**Figure 1**, A and B). Seven BA subspecies had serum bile acid (sBA) concentrations at or above the detection limits of the assay and these were mainly primary BA. Total serum BA concentrations in undernourished children were significantly higher than the healthy controls at the age of 3–6 mo (fold change = 1.4, *P* = 0.005) and also at the age of 9 mo (fold change = 1.3, *P* = 0.004). Of note, at the age of 3–6 mo, 75% (*n* = 254) of the undernourished children had a total sBA concentration well above the 8 μmol/L upper limit of the normal range for the assay, and higher than the reported Pakistani normal range (6.52 ± 0.29 μmol/L) (26–28). Aged 9 mo, elevated sBA concentrations persisted, being evident in 64% (*n* = 190) of these children and consistent with subclinical chronic liver dysfunction and/or cholestasis.

**FIGURE 2:**
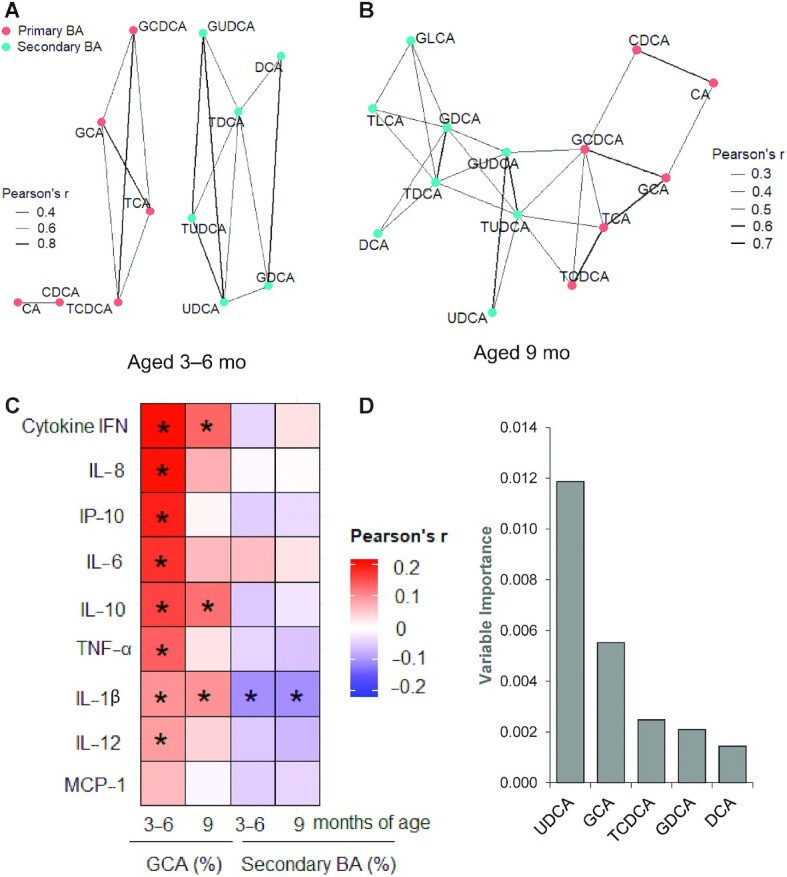
Association of BA subspecies and correlation to cytokines of Pakistani undernourished and healthy children aged between 3 and 6 mo, and 9 mo. (A–B) Correlation network analysis of serum BA subspecies concentration at the age of 3–6 mo (*n* = 389), and 9 mo (*n* = 342). GLCA and TLCA were not included in the 3–6 mo plot due to their extremely low concentration in the sample. (C) Correlation of GCA (%) in serum and proinflammatory cytokines (*n* = 384 and 339 aged 3–6 mo and 9 mo, respectively). (D) Conditional random forest (CRF) model identified the top 5 important BA in predicting height-for-age Z score at 24 mo (linear growth) using the 9 mo data set consisted of 277 Pakistani children. BA, bile acid; CA, cholic acid; CDCA, chenodeoxycholic acid; DCA, deoxycholic acid; GCA, glycocholic acid; GCDCA, glycochenodeoxycholic acid; GDCA, glycodeoxycholic acid; GLCA, glycolithocholic acid; GUDCA, glycoursodeoxycholic acid; LCA, lithocholic acid; TCA, taurocholic acid; TCDCA, taurochenodeoxycholic acid; TDCA, taurodeoxycholic acid; TLCA, taurolithocholic acid; TUDCA, tauroursodeoxycholic acid; UDCA, ursodeoxycholic acid.

When individual BA profiles were examined in detail, the concentration of GCA was significantly elevated in undernourished children compared with Pakistani well-nourished controls. The concentration of GCA was 2.6- and 2.0-fold greater at the age of 3–6 and 9 mo, respectively, (both *P* <0.001) than the local Pakistani control group, indicating GCA as a potential biomarker for early growth faltering. The mean serum concentration of the other major conjugated primary BA, GCDCA, was also significantly higher at the age of 3–6 mo (fold change = 1.2, *P* = 0.04) and 9 mo (fold change = 1.3, *P* = 0.003) in undernourished cases compared with well-nourished controls. We observed a significant negative correlation between HAZ and the relative proportion of GCA, expressed as a percentage of the total serum BA in samples at the age of 3–6 and 9 mo and prior to any nutritional intervention (**Table 3**). A correlation network analysis of the serum BA data revealed a relation among the individual BA species with 2 distinct communities/subgroups of BA evident: one subgroup consisting of primary BA and another mainly secondary BA (**Figure 2**, A and B). This subnetwork structure indicates the relation and biotransformation of these 2 groups in BA metabolism. The concentrations of secondary BA in serum were extremely low for these children, at both time points, with many samples having concentrations below the LLOQ of the assay. When expressed as a proportion of the total serum BA, secondary BA accounted for only 1.13 ± 0.14% (mean ± SEM) at 3–6 mo but increased as the children became older, accounting for 1.97 ± 0.15% at the age of 9 mo – these differences were not clinically significant. Therefore, we combined all the measured secondary BA concentrations and investigated these as a single biomarker.

**TABLE 3:** Correlation between serum BA biomarkers and growth and measured EED biomarkers among Pakistani undernourished and healthy children aged between 3 and 6 mo and 9 mo^1^

			GCA (%)			Secondary BA (%)		
Growth and EED markers	Age	Total no.	Pearson's r	Coefficient range (95% CI)	*P* value	Pearson's r	Coefficient range (95% CI)	*P* value
HAZ	3–6 mo	384	–0.252	(–0.343, –0.155)	<0.001	–0.054	(–0.154, 0.046)	0.29
	9 mo	338	–0.295	(–0.389, –0.194)	<0.001	0.089	(–0.018, 0.194)	0.10
WHZ	3–6 mo	384	–0.195	(–0.289, –0.096)	<0.001	–0.068	(–0.167, 0.032)	0.18
	9 mo	338	–0.167	(–0.268, –0.061)	0.002	0.023	(–0.084, 0.130)	0.67
AGP	3–6 mo	389	0.327	(0.235, 0.414)	<0.001	–0.031	(–0.131, 0.069)	0.54
	9 mo	340	0.226	(0.122, 0.325)	<0.001	–0.074	(–0.180, 0.033)	0.18
IGF–I	3–6 mo	386	–0.152	(–0.248, –0.052)	0.003	–0.107	(–0.205, –0.007)	0.04
	9 mo	338	–0.030	(–0.137, 0.078)	0.59	0.120	(0.012, 0.224)	0.03
Prealbumin	3–6 mo	389	0.037	(–0.065, 0.138)	0.47	0.193	(0.093, 0.289)	<0.001
	9 mo	339	–0.085	(–0.194, 0.027)	0.13	0.163	(0.053, 0.269)	0.004
Urine claudin-15	3–6 mo	384	0.123	(0.022, 0.221)	0.02	0.077	(–0.024, 0.177)	0.14
	9 mo	339	0.078	(–0.029, 0.184)	0.15	0.080	(–0.026, 0.186)	0.14

1 Dependent on the time of sample collection, monthly anthropometry data that were collected at the closest months of age were used. AGP, α-1 acid glycoprotein; BA, bile acid; EED, environmental enteric dysfunction; GCA, glycocholic acid; HAZ, height-for-age Z score; IGF-I, insulin-like growth factor I; WHZ, weight-for-height Z score.

Among blood and fecal EED biomarkers (29) that were monitored (Table 1 and **Supplemental Table 3**), AGP, IGF-I, and urinary claudin-15 showed significant correlations with linear growth. Analysis of the serum GCA concentrations, expressed as a percentage of the total sBA (%GCA) showed a positive association with AGP, a marker for acute and chronic inflammation, and claudin-15 at the age of 3–6 mo (Table 3). Serum GCA also correlated significantly with proinflammatory cytokines, i.e. cytokine IL-1β at both assessed time points. IL-1β is a key cytokine involved in both chronic and acute inflammation in the intestine and is mainly activated through innate activation of monocytes (30, 31). GCA concentrations positively correlated with most other measured proinflammatory cytokines (IFN-γ, IL-10, IL1-β, IL-6, IL-12, TNF-α) and chemokines (monocyte chemoattractant protein 1 [MCP-1], IL-8, interferon-inducible protein 10 [IP-10]) (Figure 2C). The proportion of secondary BA positively correlated with prealbumin and IGF-I at the age of 9 mo (Table 3). To find the potential predictive relation between BA biomarkers and long-term growth outcome at the age of 24 mo, we also correlated GCA and secondary BA with HAZ and WHZ. At the age of 9 mo, BA biomarkers significantly correlated with linear growth (HAZ) of the child at the age of 24 mo (**Supplemental Table 4**). Using a conditional random forest (CRF) model, the top BA biomarkers at the age of 9 mo that were predictive of linear growth at the age of 24 mo were UDCA, GCA, TCDCA, GDCA, and DCA (Figure 2D). The random forests algorithm does not provide directional relation per se. However, we obtained the directionality from a multiple linear model with adjustment for initial HAZ, WHZ, and WAZ (**Supplemental Table 5**). Among these BA subspecies, directionality of UDCA, DCA, and GUDCA were positive to HAZ at 24 mo, whereas GCA was negatively correlated. Results of the corresponding top BA biomarkers for predicting WHZ and WAZ outcomes are summarized in **Supplemental Figure 2**.

### BA dysregulation associates with histopathological severity in EED children

**FIGURE 3:**
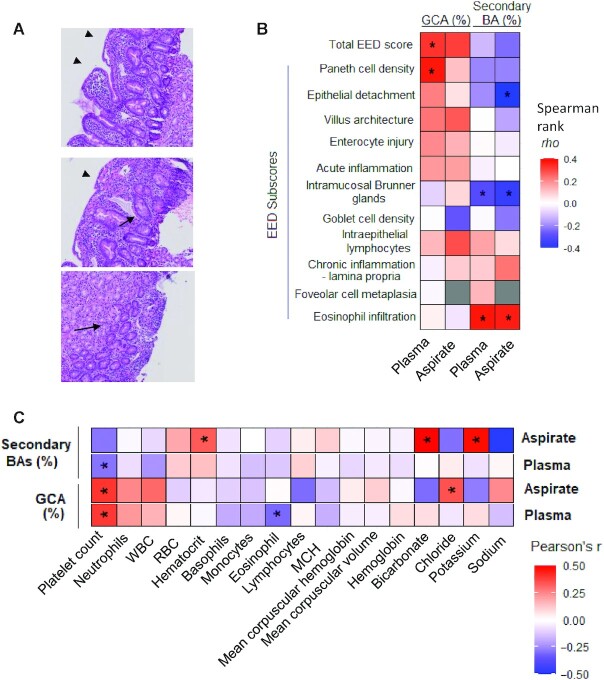
Histopathological analysis of EED children and association to plasma and duodenal aspirate BA composition. (A) Representative hematoxylin and eosin staining (H&E) duodenal histology from an undernourished EED child. Arrowhead indicates villous blunting, short arrow indicates Paneth cell, and long arrow indicates intramucosal Brunner gland hyperplasia. (B) Correlation of percent secondary BA and GCA in plasma and duodenal aspirates (*n* = 63 EED children) with histopathological EED scores. (C) BA profile associates with clinical investigation blood test results in Pakistani EED children. Pearson correlation of GCA and secondary BA (%) in duodenal aspirate (*n* = 39) and plasma (*n* = 55) to clinical investigation lab results of Pakistani EED children. *P <0.05. BA, bile acid; EED, environmental enteric dysfunction; GCA, glycocholic acid; MCH, mean corpuscular hemoglobin concentration; RBC, red blood cell count; WBC, white blood cell count.

Duodenal biopsy samples from Pakistani EED children were scored based on acute and chronic inflammation, villus architecture, secretory cells, enterocyte injury, epithelial detachment, and others (2). Representative hematoxylin and eosin staining (H&E) duodenal histology from undernourished EED children are shown in **Figure 3**A. The EED cases demonstrated intramucosal Brunner gland hyperplasia and villous blunting, and more pronounced Paneth cell depletion (**Supplemental Figure 3**). Correlations between potential BA biomarkers including GCA and secondary BA and the total EED histological scores are summarized in **Table 4** and Figure 3B.

**TABLE 4:** Correlation between BA composition and total histopathological EED score among Pakistani children evaluated by endoscopy^1^

Sample	Children in subgroup no.	Spearman's *rho* (*r_s_*)	Coefficient range (95% CI)	*P* value
Correlation total histopathological EED score and GCA (%)				
Plasma	55	0.324	(0.064, 0.543)	0.02
Duodenal aspirate	39	0.307	(–0.009, 0.568)	0.06
Correlation total histopathological EED score and secondary BA (%)				
Plasma	55	–0.121	(–0.374, 0.149)	0.38
Duodenal aspirate	39	–0.237	(–0.514, 0.085)	0.15

1 Spearman rank correlation performed for correlation of histopathological EED score of Pakistani EGD children, secondary BA (%) and GCA (%). BA, bile acid; EED, environmental enteric dysfunction; EGD, esophagogastroduodenoscopy; GCA, glycocholic acid.

### BA profiles of plasma and duodenal aspirate samples of Pakistani EED children

The clinical features of Pakistani EED children are summarized in **Supplemental Tables 6** and **7**. To overcome the limitation of lacking EGD samples from local healthy Matiari controls (as it was deemed not ethically possible to perform endoscopy on well-nourished infants), we evaluated the histological composite score by dividing the EED children into 2 subgroups according to histological severity, those below the 50th percentile (whose histological score ≤median score, *n* = 33) and those above the 50th percentile (whose histological score >median score, *n* = 30).

A total of 41 duodenal aspirates were successfully collected (2 samples had undetectable concentrations of total bile acids and were therefore excluded from data analysis) and 55 plasma samples were analyzed. It was notable that dysregulated BA metabolism was more pronounced in the cases of EED children with a histological score above the 50th percentile compared with those below the 50th percentile (**Supplemental Figure 4**A and B). Notably, in the aspirates, GCA was present in marginally significant greater proportions (*P* = 0.051), and conjugated secondary BA, i.e., GDCA, and GUDCA, were found in lower proportions in the severe EED cases. It was observed that HAZ and WAZ were worse in children with severe EED scores but not statistically significant (Supplemental Table 2). Importantly, a marginally significant decrease in secondary BA (*P* = 0.088) was observed in the duodenum of more severe EED children (**Supplemental Figure 5A**) and delineated a potentially inactive BA transformation in the gut of EED patients. The mean plasma sterol-C4 concentration, a surrogate marker for bile acid synthesis rate, in the Pakistani EED cohort was not statistically significant between mild and severe groups (*P* = 0.75) (Supplemental Figure 5B).

Although unconjugated BA in duodenal aspirates in EED children were negligible (<1%), by contrast, plasma concentrations of unconjugated BAs were significantly higher. However, there was no significant difference in the proportions of conjugated BA in Pakistani EED children (*P* = 0.37 and 0.44, respectively) between the 2 subsets (**Supplemental Figure 6A**).

The calculated HI_BA_ of duodenal aspirates increased in severe EED children compared to children with less severe disease. We also observed a similar increase of HI_BA_ in plasma but differences were not statistically significant (Supplemental Figure 6A). As cholestasis is associated with fat malabsorption, bomb calorimetry was performed on a single stool specimen from EED children to provide a readout of the intestinal absorption and fecal energy (32) (Supplemental Figure 6B). It showed that GCA (%) positively correlated, whereas secondary BA (%) and HI_BA_ in plasma and duodenal aspirates negatively correlated, with fecal energy wasting; however, this was not statistically significant.

**FIGURE 4:**
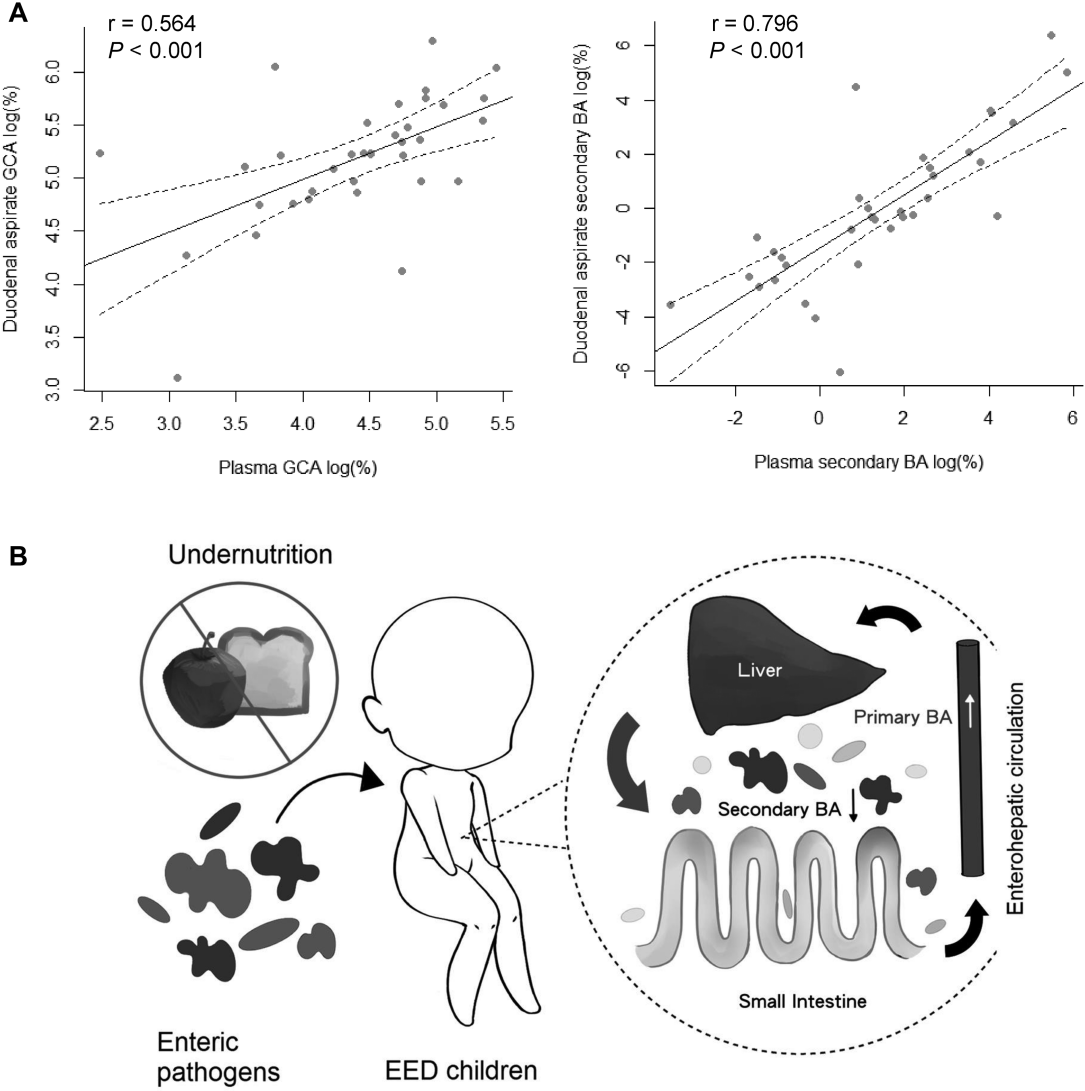
Correlation of plasma and duodenal aspirate GCA and secondary BA and their potential role as a disease-relevant signature in EED. (A) Correlation of percent GCA and secondary BAs in plasma and duodenal aspirates of EED children (*n* = 35). (B) Schematic illustration of BA signatures in undernourished EED children. BA, bile acid; EED, environmental enteric dysfunction; GCA, glycocholic acid.

The correlations between BA biomarkers and investigated clinical blood results are summarized in Figure 3C. Of note, plasma BA composition at the time of EGD (**Figure 4**A) showed a similar qualitative pattern to that of duodenal aspirates. There was a high correlation between the duodenal aspirate and plasma BA composition when bile acids were expressed as (%) total secondary BA (Pearson's r = 0.796) and GCA (Pearson's r = 0.564), indicating plasma BA composition reflects duodenal BA composition. We observed a significant correlation between plasma GCA (%) and the total EED histopathological score, and a negative correlation in secondary BA (%) in both plasma and duodenal aspirates. In particular, plasma GCA significantly correlated with Paneth cell depletion and the proportion of secondary BAs significantly correlated with intramucosal Brunner gland scores. The proportion of GCA in plasma and duodenal aspirates positively correlated with platelet and white blood cell counts, and with markers of systemic inflammation, whereas the proportion of plasma secondary BA negatively and significantly correlated with platelet count.

## Discussion

The goals of this study, which comprised 416 Pakistani children (365 undernourished and 51 age-matched well-nourished controls) prospectively followed over 2 y were to determine the extent to which: 1) bile acid metabolism is altered in undernourished children and *2*) if measures of the serum and duodenal BA metabolome could reveal specific biomarkers of EED pathology to predict later growth outcome. A targeted metabolomics approach used tandem MS to profile the major conjugated and unconjugated sBA. Sulfated BA, which are mainly excreted in urine, were not measured due to their relatively low concentrations in the serum (33). Our serial BA measurements in these infants suggest undernutrition and EED-associated bile acid dysregulation: *1*) occurs earlier in childhood than previously appreciated, *2*) precedes moderate acute malnutrition, and *3*) is not geographically restricted to sub-Saharan Africa. Furthermore, a remarkable finding was the high proportion of undernourished children with persistently elevated total sBA concentrations consistent with liver dysfunction.

SBA reflect the balance between intestinal input and hepatic extraction and are influenced by changes in hepatic synthesis and secretion as well as intestinal metabolism by the microbiome (34). The concentration and composition of BA in serum provides a sensitive indicator of the extent of hepatic dysfunction, of cholestasis, and of small bowel bacterial overgrowth (35–37). Therefore, disturbances in BA synthesis, secretion, or intraluminal bacterial metabolism can have a profound effect on lipid absorption and growth, as is evident in patients that have neonatal and infantile cholestasis, in genetic disorders of bile acid synthesis (38), and in cases of increased fecal bile acid loss (39). Our findings of disrupted BA homeostasis in undernourished children as young as 3–6 mo has implications for the absorption of preventive lipid-based nutrient supplements beyond the age of 6 mo in this and other settings. Further, the persistence of this disrupted homeostasis in children with refractory wasting provides insights into the judicious use of high lipid-based nutrient supplements to reverse moderate- and severe-acute malnutrition (40).

The primary bile acids CA and CDCA are secreted in bile almost exclusively (>99%) in a conjugated form, mainly as glycine and taurine conjugates. These primary bile acids undergo an initial intestinal hydrolysis by bacterial bile salt hydrolases and are then dehydroxylated by bacterial 7α-dehydroxylase to form the secondary bile acids, DCA and LCA (41), or further metabolized by epimerases (42) (Figure 1C). A remarkable finding from this study was the high proportion (>70%) of the undernourished children with total sBA concentrations, predominately conjugated, well above the upper limit of the normal range and consistent with mild cholestasis. When individual BA species were compared, serum GCA was found to be consistently elevated in undernourished children when compared with the cohort of Pakistani well-nourished, age-matched controls, and seemingly a useful biomarker for growth faltering. Although BA metabolism has been studied in the context of EED and in undernourished children in Malawi (11, 12), the findings are inconsistent, perhaps due to differences in the severity of undernutrition between the 2 Malawian cohorts. Total sBA, GCA, and GCDCA were reported to be increased in older Malawian children (age range 6–60 mo) with SAM (12), however, total sBA were ∼12% lower in a separate cohort of children with EED versus children without EED (11). Thus, our findings in undernourished Pakistani infants are more concordant with those from Malawian children with SAM, whose mean age was greater (24.8 ± 11.7 mo) and whose severity of undernutrition required hospitalization. In our study, elevated total BA concentrations were sustained from up to the age of 9 mo, suggesting disrupted bile acid homeostasis occurs prior to the onset of clinically significant ponderal and linear growth faltering. Indeed, such high total sBA concentrations, with low concentrations of the secondary bile acid, DCA, is indicative of significant cholestasis (35–37), as defined by impaired organic anion transport and liver dysfunction, and not because of any global differences in normal ranges (27, 28). This was an unexpected finding, and as liver function tests were never performed at the time of blood collection, it is not possible to know whether serum transaminases were also elevated. These findings, which point to subclinical cholestasis, now make a strong case for routinely performing serum liver function tests (LFTs) on undernourished children with suspected EED. Although these children did not have obvious jaundice we speculate that: *1*) elevations in sBA indicate canalicular secretion of BA into bile may be compromised and *2*) intraluminal bile acid concentrations may be below the critical micellar concentration (CMC) required for efficient lipid absorption, with consequent adverse effects on growth.

Uniquely in our study, the collection of duodenal aspirates and biopsies in those children unresponsive to nutritional intervention allowed for direct comparisons of plasma and duodenal bile acid profiles and correlations of bile acid composition with EED histopathology. It is not possible to accurately determine biliary bile acid concentrations because samples were not from the common bile duct and were not ‘pure bile,’ but rather bile diluted by gastric, pancreatic, and duodenal secretions. This makes concentrations difficult to interpret and typically leads to large interindividual variations in concentrations, as was evident from our data. For this reason, BA composition in such samples is typically expressed as percent composition for the individual species. Nevertheless, it was observed that the correlation trends were similar when absolute concentrations of BA were used (data not shown) and this information defines the composition of the BA pool.

Due to ethical constraints and the rarity of upper endoscopy performed in healthy young children living in Pakistan, comparison of duodenal aspirate data for the EED cases and a healthy Pakistani control group was not possible. To circumvent this problem, we retrospectively compared the bile acid profiles in those EED patients ranked above the 50th percentile to those below the 50th percentile based on the composite histological EED score even allowing for the limitation that the median EED composite score may not be perfect for the stratification of subgroups. Nevertheless, it provided a practical metric to evaluate the relevance of biomarkers in the absence of a healthy control group of locally age-matched children.

The finding of an association between secondary BA deficiency and EED is consistent with the hypothesis that the gut microbiome of EED children may also be compromised. Nutritional deficiency and enteropathogen exposure in EED lead to a suboptimal microbial community and injured small intestine (3, 4). In the gut-liver axis, host metabolism and gut microbiome crosstalk, partly through signaling molecules such as BA, modulate and influence each other (Figure 4B). In children with EED, primary BA were elevated in blood and secondary BA decreased in duodenal aspirates. Secondary BA depletion in the gut can result in gut microbiome changes and vice versa.

There are many reports on the role of BA in the maintenance of microbiota homeostasis and on the mucosal immune system in the intestine (14, 43). Duodenal dysbiosis in stunted Bangladeshi children with EED has been reported (19). Secondary BA deficiency in the duodenum of EED children could correlate with imbalance of the duodenal microbiome. Interestingly, the proportions of GCA positively, and secondary BA negatively, correlated with Paneth cell depletion in EED children (Figure 3B) suggesting possible pathogenic mechanisms involving altered gut microbiota. In a separate publication of this SEEM it was reported that EED cases had more pronounced Paneth cell depletion when compared with a cohort of celiac patients (20). Our results show that the proportions of serum GCA positively correlated with most measured proinflammatory cytokines and chemokines in early infancy. This raises the possibility of treating EED patients by targeting and manipulating BA composition through probiotics, prebiotics, synbiotics, or microbiome-directed diets (44). Animal studies have shown oral BA administration markedly alters the microbiome (45). Fecal bile acid analysis may reveal differences in the microbiome of undernourished children and those with EED (46) but feces (and urine) from undernourished children were not analyzed in our study. The development of a more simplistic assay for measuring GCA for use as a potential biomarker for growth outcomes in resource-restricted labs may be possible in the future.

The main limitations of such a complex and challenging study include the focus on only Pakistani children, so it is unclear if our findings generalize to other undernourished populations with EED, and the lack of a control group because performing upper endoscopy in healthy local Pakistani controls was not ethically justified. Despite these limitations, our findings reveal distinct BA biomarkers relevant to EED pathology. Previous studies have shown some heterogeneity in EED biomarkers. In a recent report, Mutasa et al. found none of the 11 putative biomarkers of EED investigated were associated with linear growth among Zimbabwean children during the first 18 mo of life (10); however, bile acids were not included. Reliable biomarkers that correlate with EED severity and growth trajectories remain an urgent need.

The strengths and unique aspects of our study include: *1*) a sizable cohort of undernourished children studied longitudinally for their bile acid metabolome, *2*) the identification of an early life specific biomarker predicting growth outcome at the age of 2 y, *3*) the findings of significant cholestasis in undernourished children, *4*) the detailed study of a subgroup of children who underwent mucosal biopsy histologic evaluation for refractory wasting to gain a mechanistic insight in EED, and *5*) establishment of a relationship between blood and duodenal BA composition to other clinical and exploratory markers of EED.

In summary, our findings reveal a potential biochemical signature for EED pathogenesis and identification of GCA as a specific biomarker for predicting growth outcome in undernourished infants, opening the possibility to explore future therapeutics that may alter the bile acid metabolome.

## Supplementary Material

nxab321_Supplemental_Files

## Data Availability

All generated data are either summarized or represented to support the findings of this work and available within the article. Raw data are available upon request.

## Notes

This work was supported by the Bill and Melinda Gates Foundation grants to SAA (OPP1138727), SRM (OPP1144149 U19 AI116491 t),the National Institute of Diabetes and Digestive and Kidney Diseases of the National Institutes of Health to SS (K23 DK117061-01A1) and FIC K43TW010697(Microbiota, Inflammation and Environmental Enteric Dysfunction).

Author disclosures: KDRS holds equity in Asklepion Pharmaceuticals LLC and is a consultant to Retrophin and Mirum Pharmaceuticals. SRM is a consultant to Takeda and also a member of the *American Society for Nutrition*. All other authors report no conflicts of interest.

Supplemental Figures 1–6, Supplemental Tables 1–7 and Supplemental Methods are available from the “Supplementary data” link in the online posting of the article and from the same link in the online table of contents at https://academic.oup.com/jn/.

XZ and KDRS are joint first authors.

Abbreviations used: AGP, α-1 acid glycoprotein; AKUH, Aga Khan University Hospital; BA, bile acid; CA, cholic acid; CCHMC, Cincinnati Children's Hospital Medical Center; CDCA, chenodeoxycholic acid; CRF, conditional random forests; DCA, deoxycholic acid; EED, environmental enteric dysfunction; EEDBI, Environmental Enteric Dysfunction Biopsy Initiative; EGD, esophagogastroduodenoscopy; GCA, glycocholic acid; GCDCA, glycochenodeoxycholic acid; GDCA, glycodeoxycholic acid; GUDCA, glycoursodeoxycholic acid; HAZ, height-for-age Z score; HI, hydrophobicity index; IGF-I, insulin-like growth factor; LC-ESI-MS, LC-electrospray ionization-MS; LCA, lithocholic acid; LFTs, liver function tests; LLOQ, lower limit of quantification; SAM, severe acute malnutrition; sBA, serum bile acid; SEEM, Study of Environmental Enteropathy and Malnutrition; TCA, taurocholic acid; TCDCA, taurochenodeoxycholic acid; TDCA, taurodeoxycholic acid; UDCA, ursodeoxycholic acid; WAZ, weight-for-age Z score; WHZ, weight-for-height Z score; sterol-C4, 7α-hydroxy-4-cholesten-3-one.
