# Supplementary material for: Bile Acid Profiling Reveals Distinct Signatures in Undernourished Children with Environmental Enteric Dysfunction
Source: J Nutr. 2021 Oct 27;151(12):3689–700. doi: 10.1093/jn/nxab321 (PMC8643614; doi:10.1093/jn/nxab321)
Supplement: nxab321_Supplemental_Files [file nxab321_supplemental_files.zip › BA_manuscript_figures_supplemental.pptx]

## Slide 1
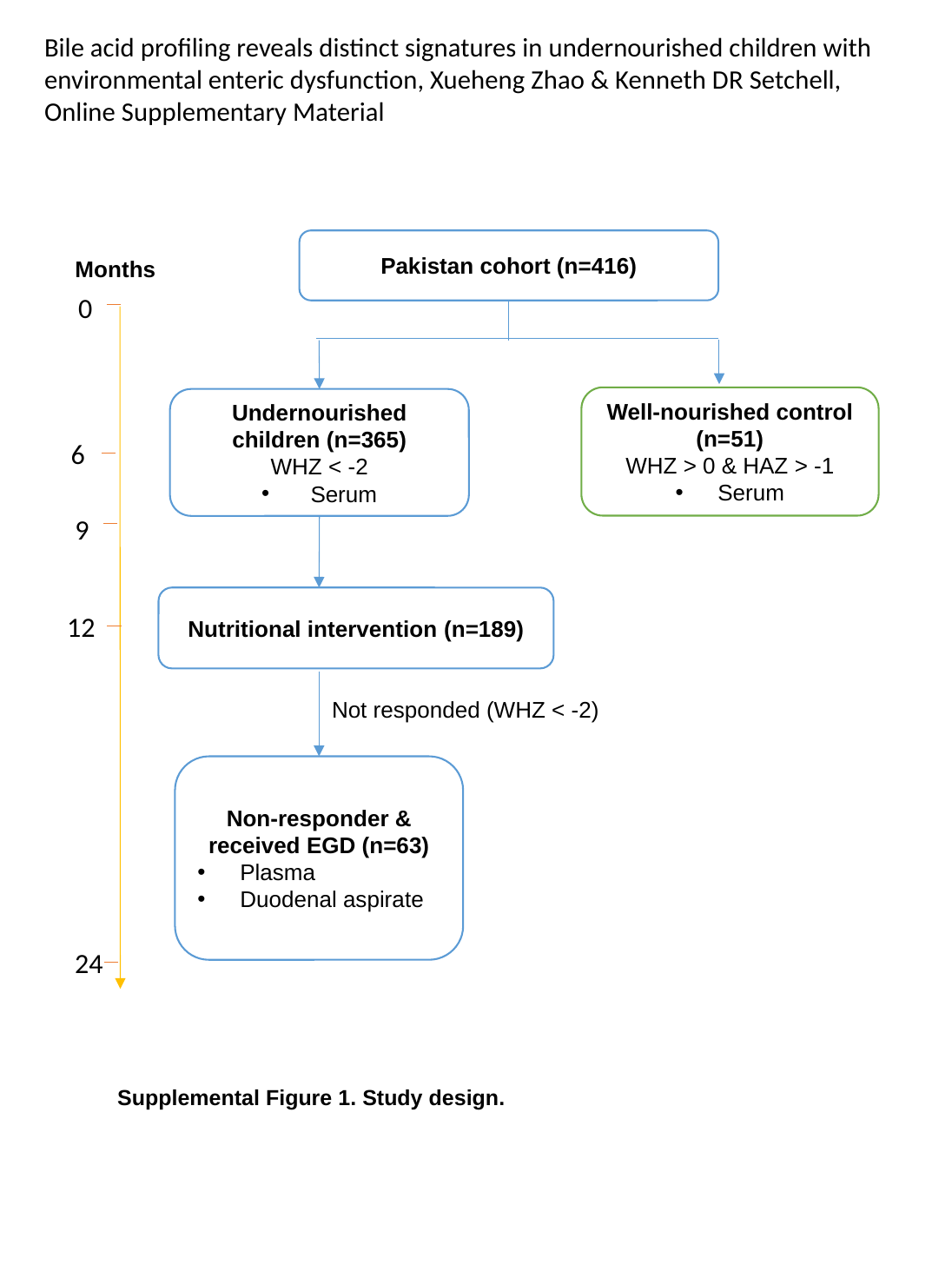

Bile acid profiling reveals distinct signatures in undernourished children with environmental enteric dysfunction, Xueheng Zhao & Kenneth DR Setchell, Online Supplementary Material
Pakistan cohort (n=416)
Months
0
Well-nourished control (n=51)
WHZ > 0 & HAZ > -1
Serum
Undernourished children (n=365)
WHZ < -2
 Serum
6
9
Nutritional intervention (n=189)
12
Not responded (WHZ < -2)
Non-responder & received EGD (n=63)
Plasma
Duodenal aspirate
24
Supplemental Figure 1. Study design.

## Slide 2
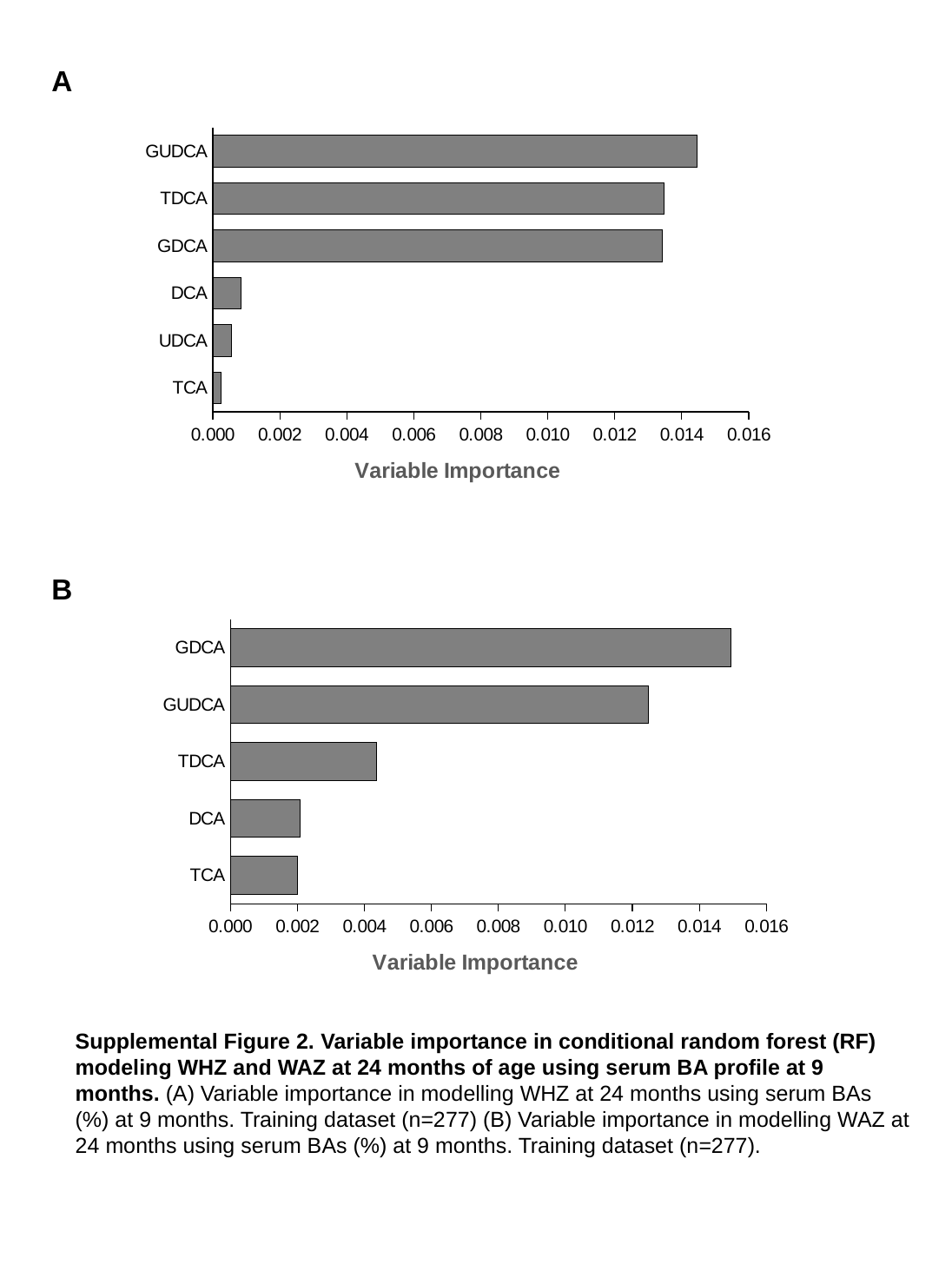

A
### Chart
| Category | Variable importance |
|---|---|
| GUDCA | 0.01445408 |
| TDCA | 0.0134823 |
| GDCA | 0.01342144 |
| DCA | 0.0008310218 |
| UDCA | 0.0005696516 |
| TCA | 0.000239391 |B
### Chart
| Category | Variable importance |
|---|---|
| GDCA | 0.0149535396 |
| GUDCA | 0.0124831529 |
| TDCA | 0.0043528952 |
| DCA | 0.0020774885 |
| TCA | 0.0020029203 |Supplemental Figure 2. Variable importance in conditional random forest (RF) modeling WHZ and WAZ at 24 months of age using serum BA profile at 9 months. (A) Variable importance in modelling WHZ at 24 months using serum BAs (%) at 9 months. Training dataset (n=277) (B) Variable importance in modelling WAZ at 24 months using serum BAs (%) at 9 months. Training dataset (n=277).

## Slide 3
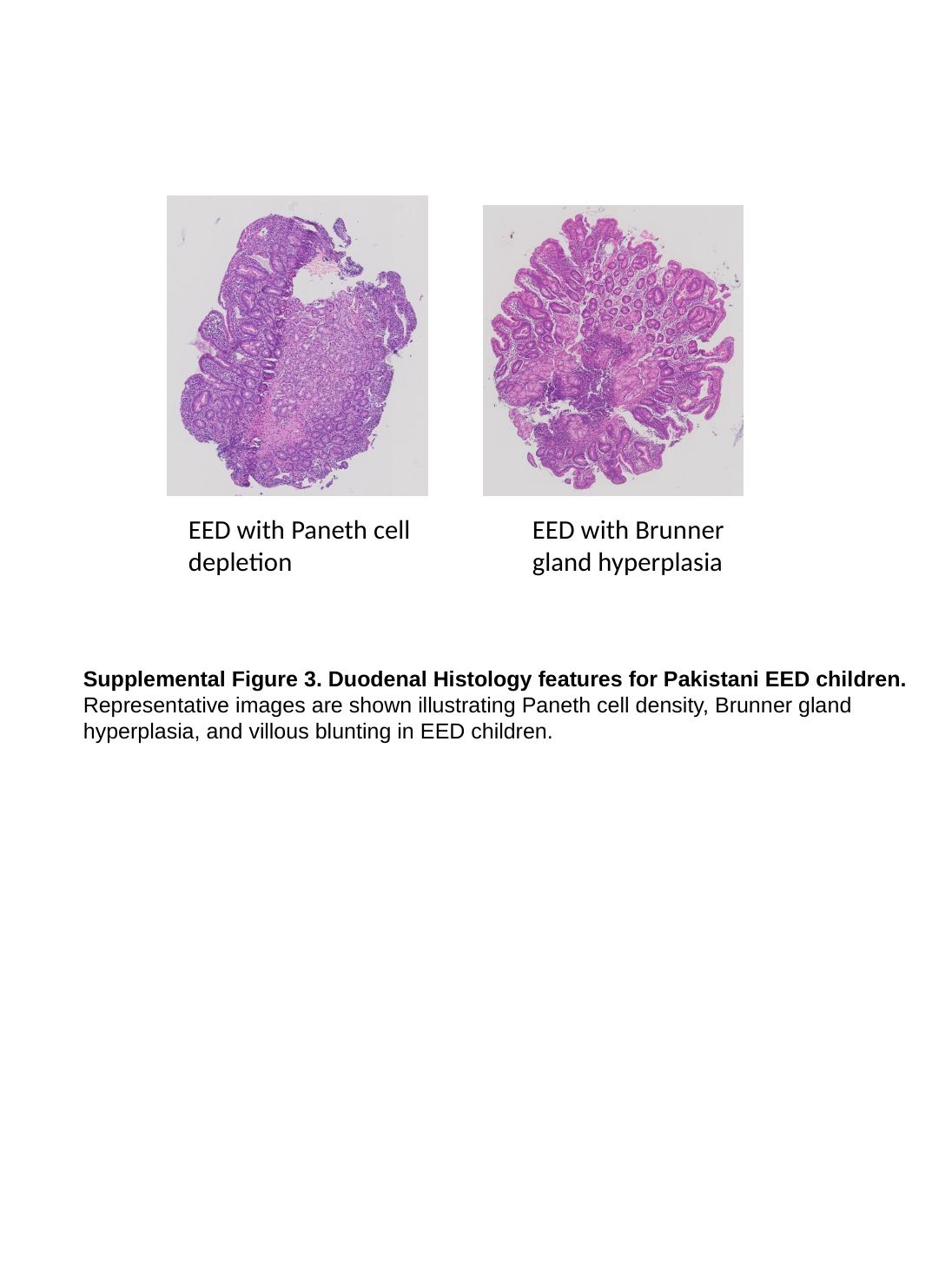

EED with Paneth cell depletion
EED with Brunner gland hyperplasia
Supplemental Figure 3. Duodenal Histology features for Pakistani EED children. Representative images are shown illustrating Paneth cell density, Brunner gland hyperplasia, and villous blunting in EED children.

## Slide 4
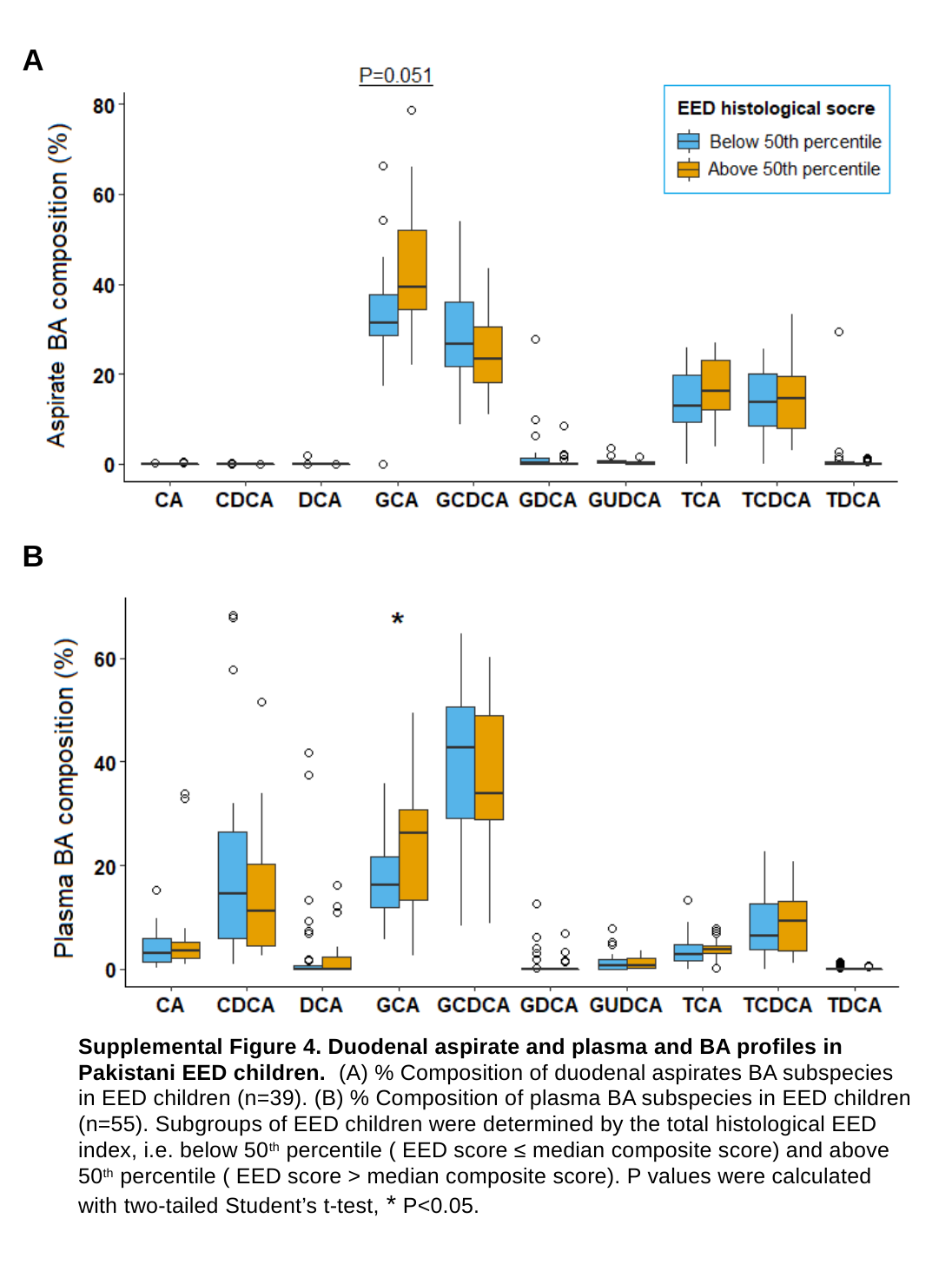

A
B
Supplemental Figure 4. Duodenal aspirate and plasma and BA profiles in Pakistani EED children. (A) % Composition of duodenal aspirates BA subspecies in EED children (n=39). (B) % Composition of plasma BA subspecies in EED children (n=55). Subgroups of EED children were determined by the total histological EED index, i.e. below 50th percentile ( EED score ≤ median composite score) and above 50th percentile ( EED score > median composite score). P values were calculated with two-tailed Student’s t-test, * P<0.05.

## Slide 5
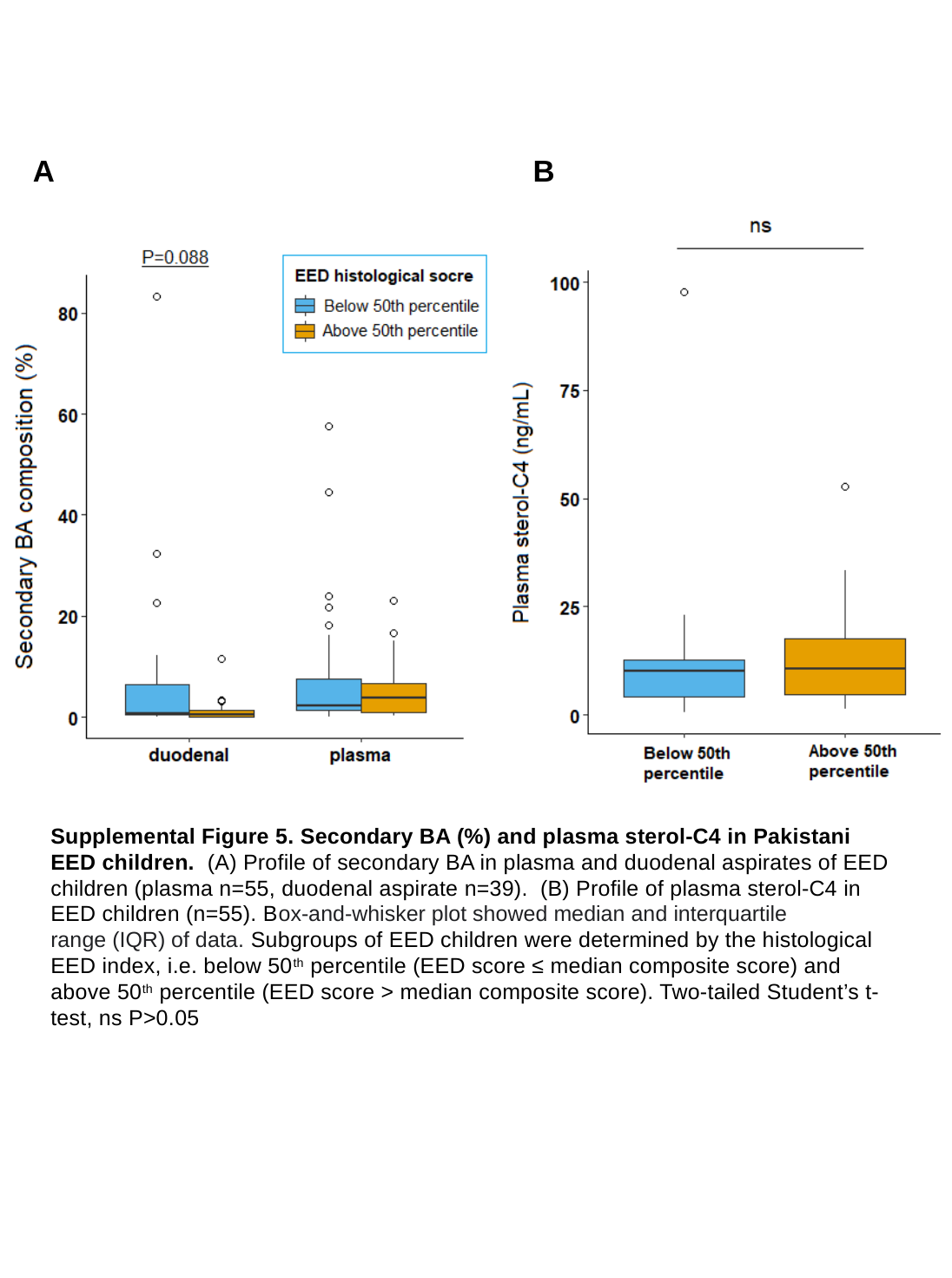

A
B
Supplemental Figure 5. Secondary BA (%) and plasma sterol-C4 in Pakistani EED children. (A) Profile of secondary BA in plasma and duodenal aspirates of EED children (plasma n=55, duodenal aspirate n=39). (B) Profile of plasma sterol-C4 in EED children (n=55). Box-and-whisker plot showed median and interquartile range (IQR) of data. Subgroups of EED children were determined by the histological EED index, i.e. below 50th percentile (EED score ≤ median composite score) and above 50th percentile (EED score > median composite score). Two-tailed Student’s t-test, ns P>0.05

## Slide 6
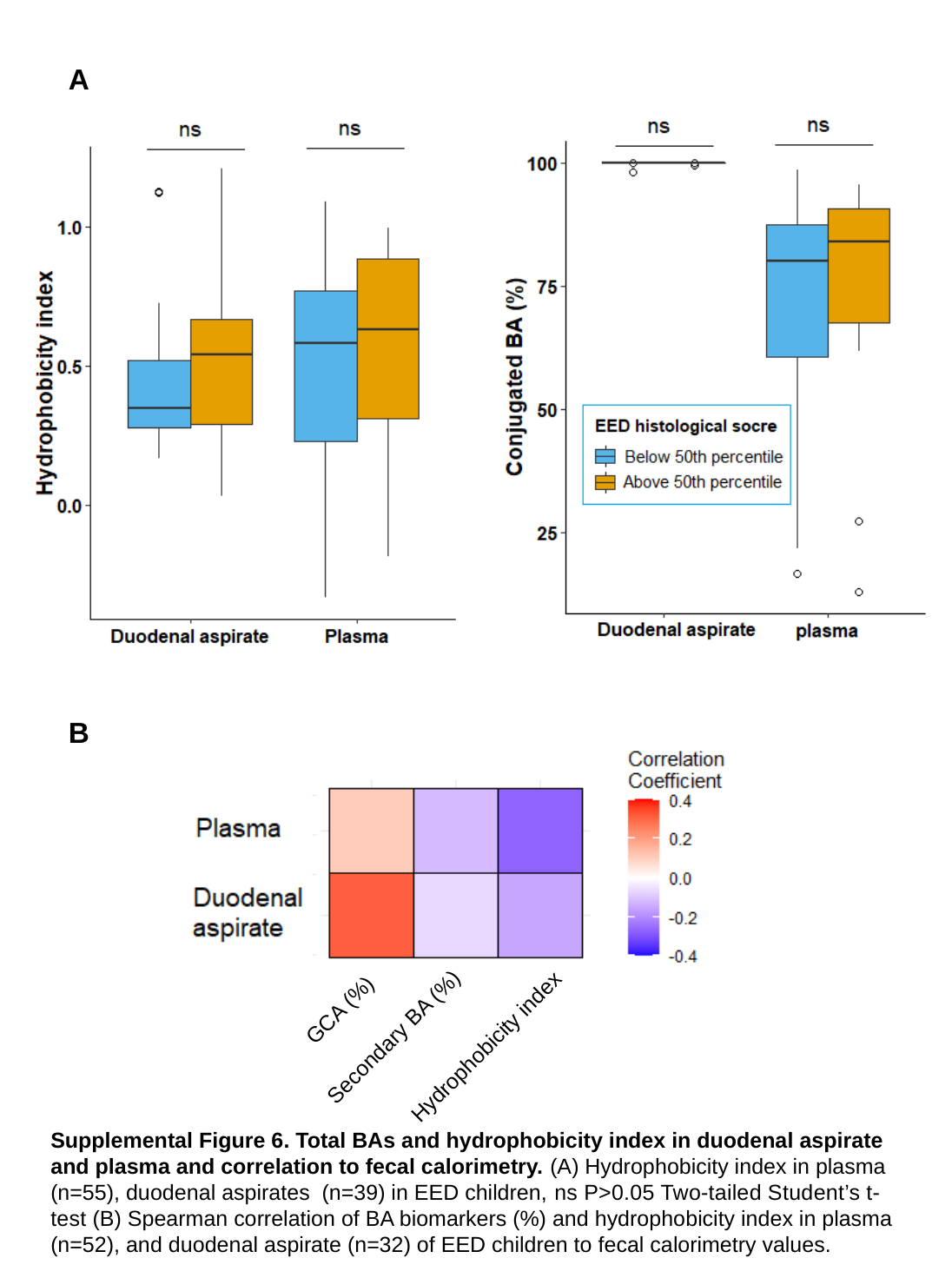

A
B
GCA (%)
Secondary BA (%)
Hydrophobicity index
Supplemental Figure 6. Total BAs and hydrophobicity index in duodenal aspirate and plasma and correlation to fecal calorimetry. (A) Hydrophobicity index in plasma (n=55), duodenal aspirates (n=39) in EED children, ns P>0.05 Two-tailed Student’s t-test (B) Spearman correlation of BA biomarkers (%) and hydrophobicity index in plasma (n=52), and duodenal aspirate (n=32) of EED children to fecal calorimetry values.
