# Supplementary material for: Bile Acid Profiling Reveals Distinct Signatures in Undernourished Children with Environmental Enteric Dysfunction
Source: J Nutr. 2021 Oct 27;151(12):3689–700. doi: 10.1093/jn/nxab321 (PMC8643614; doi:10.1093/jn/nxab321)
Supplement: nxab321_Supplemental_Files [file nxab321_supplemental_files.zip › BA_manuscript_supplemental_materials.docx]

**Supplemental Table 1.** Histological score criteria of EED

| **Feature** | **Grade** | **Description** |
| --- | --- | --- |
| Acute inflammation | 0 | No PMNs observed, or only PMNs in lamina propria with no infiltration of epithelium by PMNs (cryptitis, villitis) |
|  | 1 | 1-2 foci of epithelial PMN infiltration or crypt microabscesses |
|  | 2 | > 2 foci of epithelial PMN infiltration or crypt microabscesses but <50% of mucosa involved |
|  | 3 | > 50% of mucosa involved by epithelial PMN infiltration |
| Eosinophil infiltration | 0 | No increase in eosinophils (highly scattered in lamina propria, no intravillus or intercryptal space with >5 eosinophils) |
|  | 1 | Increased eosinophils (intravillus or intercryptal space with >5 eosinophils) involving < 50% of mucosa, with no eosinophilic crypt microabcesses |
|  | 2 | Increased eosinophils (intravillus or intercryptal space with >5 eosinophils) involving > 50% of mucosa, or up to 1 focus of eosinophilic epithelial infiltration or crypt microabcesses per mucosal fragment |
|  | 3 | >2 foci of eosinophilic epithelial infiltration or crypt microabcesses in any mucosal fragment |
| Chronic inflammation-lamina propria | 0 | No qualitative increase in mononuclear inflammatory cells (MIC) in lamina propria. Majority of villus bases contain <3 MIC across, on average. |
|  | 1 | Increased MIC, based on villus base displaying 3-5 MIC across, on average. |
|  | 2 | Increased MIC, based on villus base displaying 6-10 MIC across, on average. |
|  | 3 | Increased MIC, based on villus base displaying >10 lymphocytes on average. |
| Intra-epithelial lymphocytes | 0 | No areas observed with epithelial/lymphocyte ratio >20% |
|  | 1 | Lymphocyte/epithelial ratio >20%, but <50%, in less than 50% of mucosa |
|  | 2 | Lymphocyte/epithelial ratio >20%, but <50%, in greater than 50% of mucosa |
|  | 3 | Lymphocyte/epithelial ratio >50% in less than 50% of mucosa |
|  | 4 | Lymphocyte/epithelial ratio >50% in greater than 50% of mucosa |
| Villus architecture | 0 | Majority of villi are >3 crypt lengths long |
|  | 1 | Villi are < 3 but > 2 crypt lengths long, in < 50% of mucosa. |
|  | 2 | Majority of villi are < 2 crypt lengths long, but > 1 crypt length long |
|  | 3 | Villi absent, or <1 crypt length long, in < 50% of mucosa. |
|  | 4 | Villi absent, or <1 crypt length long, in > 50% of mucosa. |
| Intramucosal Brunner glands | 0 | None observed |
|  | 1 | One or two foci, none involving more than 5 crypt bases |
|  | 2 | 3-5 foci, none involving more than 5 crypt bases |
|  | 3 | > 5 foci, or any area of intramucosal Brunner glands involving >5 crypt bases |
| Foveolar cell metaplasia | 0 | Not observed |
|  | 1 | 1-2 villus tips involved |
|  | 2 | 3-5 villus tips involved |
|  | 3 | > 5 villus tips involved |
| Goblet cell density | 0 | Most villi contain >10 goblet cells |
|  | 1 | Goblet cells <10/ villus, involving < 25% of mucosa |
|  | 2 | Goblet cells <10/ villus, involving 25-50% of mucosa |
|  | 3 | Goblet cells <10/ villus, involving >50% of mucosa |
| Paneth cell density | 0 | >5 Paneth cells/ crypt, on average |
|  | 1 | 2-4 Paneth cells/ crypt, on average |
|  | 2 | <2 Paneth cell/crypt, involving <50% of crypt bases |
|  | 3 | <2 Paneth cell/crypt, involving >50% of crypt bases |
| Enterocyte injury | 0 | Majority of enterocytes (90%) show tall columnar morphology |
|  | 1 | Enterocytes show low columnar (<2:1 L:W ratio), cuboidal or flat morphology, in < 50% of mucosa |
|  | 2 | Enterocytes show low columnar (<2:1 L:W ratio), cuboidal or flat morphology, in > 50% of mucosa |
|  | 3 | Any area of mucosal erosion/ulceration |
| Epithelial detachment | 0 | Complete coverage of mucosal surface by epithelial cells |
|  | 1 | Surface epithelium missing or detached from <25% of mucosa |
|  | 2 | Surface epithelium missing or detached from 25-50% of mucosa |
|  | 3 | Surface epithelium missing or detached from 51-75% of mucosa |
|  | 4 | Surface epithelium missing or detached from >75% of mucosa |

**Supplemental Table 2.** Demographic and growth outcome^1^ of Pakistan enrolled children at the time of EGD^2^

| EED histopathological score | Number of children | Age | Female | WHZ | HAZ | WAZ |
| --- | --- | --- | --- | --- | --- | --- |
| ≤ 50 percentile | 33 | 20.42 ±3.47 | 14 (42%) | -2.23 ±0.77 | -3.03 ±1.11 | -3.17 ±0.87 |
| >50 percentile | 30 | 17.40 ±3.46 | 5 (17%) | -2.24 ±0.76 | -3.23 ±1.31 | -3.27 ±1.03 |

^1^At the time of UGI endoscopy/EGD, different for each participant, data are represented as mean ± SEM

^2^EGD, esophagogastroduodenoscopy; HAZ, height-for-age Z score; WAZ, weight-for-age Z score; WHZ, weight-for-height Z score.

**Supplemental Table 3:** Correlation of clinical investigation of Pakistani enrolled children to length (HAZ) and wasting (WHZ) at 3-6 months and 9 months of age^1,2^

|  | Age/Sample source | Children total  no. | *HAZ* | | | *WHZ* | | |
| --- | --- | --- | --- | --- | --- | --- | --- | --- |
|  |  |  | Pearson Correlation Coefficient | Coefficient range (95% CI) | P- Value | Pearson Correlation Coefficient | Coefficient range (95% CI) | P- Value |
| *AGP* | Between 3 and 6 month^1^ | 389 | -0.162 | (-0.257, -0.064) | 0.001 | 0.004 | (-0.095, 0.104) | 0.93 |
|  | 9 month | 340 | -0.301 | (-0.394, -0.201) | <0.001 | -0.186 | (-0.287, -0.082) | <0.001 |
| *IGF-1* | Between 3 and 6 month | 386 | 0.361 | (0.271, 0.445) | <0.001 | 0.265 | (0.170, 0.355) | <0.001 |
|  | 9 month | 338 | 0.261 | (0.159, 0.358) | <0.001 | 0.273 | (0.172, 0.369) | <0.001 |
| *CRP* | Between 3 and 6 month | 389 | -0.053 | (-0.152, 0.047) | 0.30 | -0.055 | (-0.154, 0.044) | 0.28 |
|  | 9 month | 340 | -0.022 | (-0.128, -0.084) | 0.68 | -0.030 | (-0.136, 0.077) | 0.58 |
| *Fecal Neopterin* | Between 3 and 6 month | 386 | 0.064 | (-0.036, 0.163) | 0.21 | 0.027 | (-0.073, 0.126) | 0.60 |
|  | 9 month | 360 | 0.016 | (-0.087, 0.119) | 0.76 | -0.006 | (-0.110, 0.097) | 0.91 |
| *Fecal Myeloperoxidase* | Between 3 and 6 month | 386 | 0.046 | (-0.054, 0.145) | 0.37 | 0.001 | (-0.099, 0.101) | 0.98 |
|  | 9 month | 359 | -0.042 | (-0.145, 0.062) | 0.43 | -0.029 | (-0.132, 0.075) | 0.59 |
| *Urine Claudin 15* | Between 3 and 6 month | 382 | -0.149 | (-0.246, -0.050) | 0.003 | -0.174 | (-0.270, -0.075) | <0.001 |
|  | 9 month | 357 | -0.138 | (-0.238, -0.034) | 0.009 | -0.205 | (--0.302, -0.103) | <0.001 |
| *Urine L/R test* | Lactulose 14 months | 240 | -0.103 | (-0.227, 0.024) | 0.11 | 2.7E-6 | (-0.127, 0.127) | 0.99 |
|  | Rhamnose 14 months | 240 | -0.050 | (-0.176, 0.077) | 0.44 | 0.003 | (-0.040, 0.188) | 0.97 |
|  | L/R 14 months | 240 | -0.071 | (-0.173, 0.055) | 0.27 | -0.009 | (-0.136, 0.104) | 0.89 |

^1^Dependent on the time of sample collection, monthly anthropometry data that were collected at the closest months of age were used

^2^AGP, Alpha-1 acid glycoprotein; CRP, C-reactive protein; HAZ, height-for-age Z score; IGF-1, insulin-like growth factor 1; L/R, lactose/rhamnose; WHZ, weight-for-height Z score.

**Supplemental Table 4.** Correlation between serum BA biomarkers at between 3 and 6 months and 9 months of age and growth outcome of Pakistan children at 24 months of age^1^

| Growth outcome at 24 months of age | Age of serum sample collected | Total  no. | *GCA (%)* | | | *Secondary BAs (%)* | | |
| --- | --- | --- | --- | --- | --- | --- | --- | --- |
|  |  |  | Pearson’s r | Coefficient range (95% CI) | P- Value | Pearson’s r | Coefficient range (95% CI) | P- Value |
| HAZ | 3-6 months | 335 | -0.065 | (-0.171, -0.065) | 0.24 | -0.014 | (-0.154, 0.046) | 0.80 |
|  | 9 months | 324 | -0.217 | (-0.318, -0.110) | 8.3e-5 | 0.139 | (0.031, 0.245) | **0.01** |
| WHZ | 3-6 months | 335 | 0.056 | (-0.051, 0.163) | 0.30 | 0.099 | (-0.008, 0.204) | 0.07 |
|  | 9 months | 324 | 0.020 | (-0.089, 0.129) | 0.72 | 0.031 | (-0.077, 0.140) | 0.57 |

^1^BA, bile acid; GCA, glycocholic acid; HAZ, height-for-age Z score; WHZ, weight-for-height Z score.

**Supplemental Table 5a**: Summary of linear model for the HAZ at 24 months^1^

| **Parameter** | **Estimate** | **95% Confidence Interval** | | **P-value** |
| --- | --- | --- | --- | --- |
| Intercept | -1.193 | -1.806 | -0.581 | 0.000 |
| Initial HAZ | 0.492 | 0.393 | 0.590 | <.0001 |
| UDCA > 0 vs 0 | 0.028 | -0.249 | 0.304 | 0.844 |
| log_GCA | -0.142 | -0.337 | 0.052 | 0.151 |
| DCA > 0 vs 0 | 0.176 | -0.326 | 0.679 | 0.490 |
| log_GUDCA | 0.035 | -0.229 | 0.298 | 0.796 |

^1^GCA, glycocholic acid; DCA, deoxycholic acid; GUDCA, glycoursodeoxycholic acid; HAZ, height-for-age Z score; UDCA, ursodeoxycholic acid.

**5b**: Summary of linear model for the WHZ at 24 months^1^

| **Parameter** | **Estimate** | **95% Confidence Interval** | | **P-value** |
| --- | --- | --- | --- | --- |
| Intercept | -0.896 | -1.130 | -0.662 | <.0001 |
| Initial WHZ | 0.244 | 0.129 | 0.359 | <.0001 |
| GDCA > 0 vs. 0 | -0.412 | -0.742 | -0.082 | 0.015 |
| log_GUDCA | 0.170 | -0.063 | 0.402 | 0.152 |
| TDCA > 0 vs 0 | -0.219 | -0.530 | 0.093 | 0.168 |
| DCA > 0 vs 0 | 0.295 | -0.276 | 0.866 | 0.310 |

^1^GDCA, glycodeoxycholic acid; TDCA, taurodeoxycholic acid; WHZ, weight-for-height Z score.

**5c**: Summary of linear model for the WAZ at 24 months^1^

| **Parameter** | **Estimate** | **95% Confidence Interval** | | **P-value** |
| --- | --- | --- | --- | --- |
| Intercept | -1.309 | -1.847 | -0.771 | <.0001 |
| Initial WAZ | 0.436 | 0.339 | 0.532 | <.0001 |
| GDCA > 0 vs 0 | -0.347 | -0.690 | -0.005 | 0.047 |
| log_GUDCA | 0.127 | -0.112 | 0.365 | 0.297 |
| DCA > 0 vs 0 | 0.248 | -0.314 | 0.809 | 0.386 |
| log_TCA | -0.083 | -0.322 | 0.156 | 0.494 |

^1^GCA, glycocholic acid; TCA, taurocholic acid; WAZ, weight-for-age Z score.

**Supplemental Table 6:** Results from Lactose/rhamnose (L/R) test of Pakistani children

|  | Age at sample  Collection (months) | Pakistani Children did not received EGD (n=188) | Undernourished children at enrollment and received EGD^2^ (n= 52) | Total Pakistani Children (n= 240) |
| --- | --- | --- | --- | --- |
| Lactose (µmol/L) | 13.8 ± 0.1 | 41.3 ± 3.3 | 42.1 ± 7.0 | 41.7 ± 3.0 |
| Rhamnose (µmol/L) | 13.8 ± 0.1 | 89.8 ± 7.0 | 88.7 ± 12.7 | 89.6 ± 6.1 |
| L/R ratio | 13.8 ± 0.1 | 0.72 ± 0.06 | 0.73 ± 0.09 | 0.72 ± 0.05 |

^1^Data are represented as mean ± SEM

^2^EGD, esophagogastroduodenoscopy.

**Supplemental Table 7.** Results from clinical investigation of blood and fecal samples of Pakistan enrolled children at the time of EGD^2^

|  | Undernourished at enrollment and received EGD (Matiari, Pakistan) n= 63 |
| --- | --- |
| Serum Sodium (mmol/L) | 134.4± 2.1^1^ |
| Serum Potassium (mmol/L) | 5.0 ± 0.5 |
| Serum Chloride (mmol/L) | 95.6± 2.2 |
| Serum Bicarbonate (mmol/L) | 19.2 ± 0.9 |
| Hemoglobin (g/dL) | 11.4 ± 1.4 |
| Hematocrit (%) | 32.5 ± 0.3 |
| R.B.C (million cells/µL) | 4.8 ± 0.07 |
| M.C.V (fL) | 67.9 ± 1.0 |
| M.C.H (g/dL) | 21.0 ± 0.4 |
| M.C.H.C (g/dL) | 30.6± 0.2 |
| WBC count (n x 1000/µL) | 14.7± 0.6 |
| Neutrophils (n x 1000/µL) | 29.7± 1.5 |
| Lymphocytes (%) | 53.8± 1.7 |
| Eosinophil (%) | 9.2 ± 1.6 |
| Monocytes (%) | 8.6 ± 0.3 |
| Basophils (%) | 0.4± 0.1 |
| Platelet count (n x 1000/µL) | 517.2± 23.6 |
| Fecal calorimetry (kcal/g) | 4.670 ± 0.110 |

^1^At the time of UGI endoscopy/EGD, different for each participant, data are represented as mean ± SEM

^2^EGD, esophagogastroduodenoscopy; M.C.H, mean corpuscular hemoglobin concentration; M.C.H.C, mean corpuscular hemoglobin concentration; M.C.V., mean corpuscular blood cell volume; RBC, red blood cell count; WBC, white blood cell count.

**Supplemental Methods**

BA profiling by ultra-performance liquid chromatography coupled with tandem mass spectrometry (UPLC-MS/MS)

The serum and plasma BA metabolome was determined by stable-isotope dilution tandem mass spectrometry after extraction of bile acids from biological samples by protein precipitation according to a fully validated assay (CCHMC SOP # PATH.CMS.1065). Specifically, the internal standards, a cocktail of 15 deuterium labeled standards were added to 20 µL serum samples. Calibrators and QC serum samples that were spiked with known concentrations of BA to charcoal stripped serum were processed following the same procedure. The samples were vortexed before adding acetonitrile. After vortexing and centrifuging at 13,400 g at 4°C, the supernatant was removed and dried under nitrogen gas. The extract was then dissolved in methanol/water (50/50 v/v) for UPLC-MS/MS analysis. Duodenal aspirate samples were extracted with a solid phase extraction (SPE) method^1^. After dilution with deionized water, duodenal aspirate samples passed through a C18-Bond Elut cartridge (Varian, Palo Alto, CA) to absorb BA after methanol activation and water rinse of the cartridge. After being washed with distilled water, BA including their conjugates were recovered by elution with methanol. The methanolic extract was evaporated to dryness under nitrogen and reconstituted in 200 µL mobile phase.

Quantitative analysis of the individual major BA in serum, i.e. TCA, TUDCA, TCDCA, TDCA, TLCA, GCA, GUDCA, GCDCA, GDCA, GLCA, CA, UDCA, CDCA, DCA, and LCA was conducted. UPLC-MS/MS with electrospray ionization (ESI) analysis was conducted on a Waters Xevo TQ-S triple quadruple mass spectrometer interfaced with an Equity UPLC system (Waters Corporation, Milford, MA). Individual bile acid species were separated on a Kinetex C18 (2.6 µm, 100 x 3.0 mm) column (Phenomenex, Torrance, CA) with gradient elution consisting of mobile phase A (20% acetonitrile/water with 10mM ammonium acetate) and mobile phase B (80% acetonitrile/water with 10mM ammonium acetate) programmed from 5% B to 100%B with a total run time of 20 min. Quality control (QC) samples were prepared at concentrations of 0.1, 0.5, 1.0, and 2.5 µg/mL for both conjugated and unconjugated BA and an additional QC sample of 20.0 µg/mL for the conjugated BA only. Intra- and inter-assay imprecision of the method for the 15 individual bile acids measured was within 20% coefficient of variance for these QC samples. The lower limit of quantification (LLOQ) of the assay was set at 0.1 µg/mL. Serum bile acid concentrations are expressed as µmol/L and the total serum bile acid concentration is represented by the sum of the individual bile acid species measured. The composition of individual BA was calculated as the percentage of the concentration of individual BA relative to the total. The ratio of primary to secondary BA was calculated as the ratio of the sum of the concentrations of CDCA and CA to the sum of the concentrations of DCA, LCA, and UDCA in all their conjugate forms.

**References**

1. Setchell KD, Worthington J. A rapid method for the quantitative extraction of bile acids and their conjugates from serum using commercially available reverse-phase octadecylsilane bonded silica cartridges. Clin Chim Acta 1982;125:135-44.
